# Editorial for Special Issue: “Airborne Microbes and Their Potential Influence”

**DOI:** 10.3390/microorganisms12020361

**Published:** 2024-02-09

**Authors:** Daisuke Tanaka, Fumito Maruyama

**Affiliations:** 1 School of Science, Academic Assembly, University of Toyama, 3190 Gofuku, Toyama City 930-8555, Toyama, Japan; 2 Center for the Planetary Health and Innovation Science (PHIS), The IDEC Institute, Hiroshima University, 1-3-2 Kagamiyama, Higashi-Hiroshima City 739-8511, Hiroshima, Japan; fumito@hiroshima-u.ac.jp

Airborne microbes, comprising a diverse range of bacteria and fungi, are a pervasive component of the atmosphere, with concentrations typically ranging from 10^2^ to 10^7^ cells per cubic meter [1]. These organisms are introduced into the atmosphere from various natural and anthropogenic sources [2]. Natural sources include oceans, where breaking waves release marine microbes into the air, and forests, where plant leaves and soil release microbial spores and bacteria. Anthropogenic activities contributing to airborne microbial populations include agricultural practices, where soil tilling and crop harvesting disperse microorganisms, and urban and industrial activities, such as construction and wastewater treatment, which release diverse microbes into the atmosphere.

Once airborne, these microbes face hostile conditions such as nutrient scarcity, desiccation, exposure to ultraviolet (UV) radiation, and fluctuations in temperature and pH levels [2]. These challenges have led to the evolution of various adaptive mechanisms among these microorganisms, making them a fascinating subject of study for microbiologists and atmospheric scientists.

The roles of airborne microbes are extensive and critical across various domains, including the following:

Public Health: They are key in understanding the transmission dynamics of infectious diseases, as certain pathogens can become airborne and pose health risks [2,3,4,5,6,7].

Agriculture: Some airborne microbes benefit plant growth, while others can cause plant diseases [8,9].

Food Processing: They play roles in fermentation processes but also pose contamination risks [4].

Pollutant Degradation: Certain microbes participate in the breakdown of environmental pollutants [10,11].

Global Climate: They can influence weather patterns and climate processes [12].

Despite their significance, a comprehensive understanding of airborne microbes remains elusive, primarily due to the complexity of their interactions with the environment and other organisms.

In this Special Issue of *Microorganisms*, we present the following four research papers and a review that offer valuable insights into this field:

A study on healthcare facilities reveals the presence and spread of pathogenic microbes in hospital settings (contribution 1). 

Research regarding the vicinity of beef cattle feedlots discusses the airborne transmission of agricultural microbes and potential health implications (contribution 2).

An investigation into 4D movie theaters uncovers their unique indoor microbial communities and their health impacts (contribution 3).

A study on urban pedestrian bridges examines the diversity and density of microbes in urban air and their potential health risks (contribution 4).

The review by Lyu et al. (contribution 5) delves into the intricate relationship between the airborne bacterial microbiome and allergic respiratory diseases, highlighting the significant impact of environmental microbes on human health.

The increasing interest in the impacts of airborne microbes extends beyond health to encompass climate studies. This interest is driven by the realization that airborne microbes can influence atmospheric processes [1,12]. Furthermore, the study of these organisms in indoor environments, where a significant portion of human life unfolds [13,14], is gaining prominence, given the potential health implications.

Looking ahead, future research needs to focus on understanding the myriad factors influencing the composition and dynamics of the airborne microbiome and unraveling its multifaceted impacts on human health, agriculture, and the global climate.
